# Health assessment of taxi drivers in the city of Tshwane

**DOI:** 10.4102/curationis.v39i1.1671

**Published:** 2016-11-30

**Authors:** Tendani S. Ramukumba, Makwena S. Mathikhi

**Affiliations:** 1 Adelaide Tambo School of Nursing, Tshwane University of Technology, South Africa

## Abstract

**Background:**

Taxi driving seems to be a strenuous occupation. There was evidence-based paucity of literature on health assessment of taxi drivers. Meanwhile taxi drivers of South Africa were burdened by communicable and non-communicable diseases including high-level exposure to injuries and criminal attacks. Health assessment of this cohort group enables mitigation to engage in appropriation of relevant interventions related to the occupational needs of taxi drivers.

**Objectives:**

The objective of the study was to conduct health assessment of taxi drivers in the city of Tshwane to identify health risk factors.

**Method:**

An exploratory, descriptive and quantitative survey was conducted and anthropometric measurements of blood pressure, body mass index and waist circumference were monitored and recorded on a convenience sample of 69 taxi drivers in Tshwane Municipality. Consent was sought from individual taxi drivers who participated in the study, while taxi rank queue marshals assisted with smooth running of the process. Data were gathered using a questionnaire. Data analysis was performed using statistical STATA II with the assistance of a statistician.

**Results:**

The study found that taxi drivers were obese, hypertensive, had type II diabetes-related risk factors, including unhealthy life style practices. The results indicate that the general health of taxi drivers impacts their occupation.

**Conclusion:**

The findings implicate that the health status of taxi operators in Tshwane was a serious concern and urgent concerted effort is needed to engage in lifestyle modification of taxi drivers. The need for health promotion and formalised occupational health services was recommended.

## Introduction

Health is a basic constitutional right for all South African citizens. In South Africa, there is evidence of a quadruple burden of disease which included communicable and non-communicable diseases as it is presented in the general population, including taxi drivers. The taxi industry mostly employs men as drivers and they are found to be experiencing the harsh realities of the burden of diseases, especially injuries caused by road accidents, which are directly related to their working environment (Norman *et al*. 2006). Furthermore, taxi drivers’ quarter system of salaries compromises the lifestyle practices. The behaviour of wanting to collect as much money as possible makes them susceptible to unhealthy lifestyle disorders such as obesity, chronic ischemic heart disease, type 2 diabetes, and HIV and AIDS – all of which could be prevented. Furthermore, Dahl *et al*. (2009) found that lifestyle-related diseases were the most common cause of hospitalisation amongst professional drivers in the transport industry because of the sedentary nature of their work which often leads to smoking, consumption of stimulants, such as coffee, coke and alcohol and lack of physical exercise. Taxi drivers in South Africa are not subjected to regular health assessment to identify health risks that may pose as occupational risk factors. The problem therefore is that there is a dearth of information on occupational health and safety risk assessment amongst taxi drivers in the city of Tshwane.

### Problem statement

Taxi drivers are exposed to stressful environments which impact the quality of life (Ramukumba 2012). There is a need to obtain the baseline health information for taxi drivers to strategise for exclusion of health risks of this cohort group. Ncama *et al*. (2013) confirmed that the taxi industry forms the backbone of the public transport industry and accounts for the bigger chunk of daily public commuting. It is therefore important to ensure that taxi drivers remain healthy to the benefit of themselves and their passengers.

The lack of evidence of health information of taxi drivers makes it difficult to even begin to plan for health and wellness interventions. Excluding health promotion strategies related to HIV and AIDS, strategies to improve healthy lifestyles are not relevant, purposeful and focused. The health risks related to the sedentary nature of their work result in specific and contextualised health problems, and the approach to health-improving strategies should be holistic and comprehensive (Peerson & Saunders 2009).

### Aims of the study

The aims of this study were to assess the status of health and wellness of taxi drivers in their working environment in Tshwane. Data collected from the participants will guide evidence-based intervention prevention and lifestyle modification.

### Background

The city of Tshwane was the setting for the study which was conducted amongst taxi drivers identified as the target population. Taxi drivers were identified by the researchers to be the most vulnerable group that do not use health facilities, yet they were faced with health challenges related to their lifestyle. Based on the constitution of South Africa, Chapter 2 – the Bill of Rights, taxi drivers are ensured of the right to fair labour relations, to an environment that is not harmful to their health or well-being and right to have access to healthcare services (South Africa 1996a:7–39). The Occupational Health and Safety Act (OHSA) section 12(C) (South Africa: 1993) requires performance of medical surveillance for four high-risk occupations which include taxi operators. Medical surveillance for drivers including taxi drivers should include the standard medical examination with a special emphasis on controlled chronic illnesses such as hypertension, diabetes and AIDS. As employees working in the transportation industry, taxi drivers have the right to regular medical surveillance to ensure their optimal health and safety.

### Trends

There is not much in the literature on assessment of taxi driver’s health status in South Africa and in Tshwane. Numerous studies have been conducted globally about health and safety environment of taxi drivers (Lim *et al*. 2010; Ueda *et al*. 1992).

#### Research objectives

The objective was to do assessment of anthropometric measurements of taxi drivers in their work environment, identify occupational health risks associated with taxi driving as an occupation and explore the need for specific occupational health services for taxi drivers.

### Definition of key concepts

Taxi driver is defined as the kombi driver who ferries commuters from one area to another (National Road Traffic Act (1996b).

Obesity is defined as abnormal or extensive fat accumulation that negatively affects health (Stenholm *et al*. 2008).

WHO defined obesity as body mass index (BMI) −30 kg/m^2^ and central obesity as a waist circumference greater than 102 cm in men and 88 cm in women.

Hypertension refers to a progressive cardiovascular (CV) syndrome arising from complex and interrelated aetiologies; therefore, blood pressure (BP) can be used as a biomarker of hypertension.

Stage 1 BP = 115/75 mmHg with early CV markers, Stage 2 = 140/90 mmHg with diffuse disease markers and Stage 3 = 140/90 mmHg–160/100mmHg with overt CVD markers (Giles *et al*. 2009).

Quality of life is an embracing multidimensional concept that can be categorised as physical, material, social and emotional well-being including development and activity of a human being (Felce & Perry 1995).

### Literature review

Around the world, work has heavy impact on health (Nelson *et al*. 2005). Furthermore, quantification of the burden of disease and injury because of selected occupational exposure could be substantially reduced through proven risk prevention strategies. Non-communicable diseases in adults vary greatly across regions. Leading risk factors are high blood pressure (HBP), tobacco smoking including secondary smokers and household air pollution. Interestingly, alcohol use was the leading cause in Eastern Europe and Andean Latin America, while in most of Asia, Latin America, North Africa, Middle East and central Europe it was HBP, and BMI increased globally with Australasia and Southern Latin America including other high-income regions (Lim *et al*. 2010). In studies published in peer reviewed psychological and medical journals, the results show that there was a good reason to be concerned about the possible detrimental effects of long work hours on health, in particular CV disease, diabetes, illnesses, leading to disability, retirement, subjectively reported physical ill health and fatigue (van der Hulst 2003).

Furthermore, a study conducted amongst taxi drivers in Chicago found that their poor health practices contributed to low risk profile (Apontaku-Onayeni *et al*. 2012). A recent study conducted in KwaZulu-Natal reported that taxi drivers practiced unsafe sexual intercourse without use or inconsistent use of condoms (Ncama *et al*. 2013). This study became more significant as taxi drivers are a relatively autonomous and hard-to-reach group, and there is proven paucity in knowledge about their health (Apontaku-Onayeni *et al*. 2012).

Ueda *et al*. (1992) in a Japanese taxi driver health study and Nasri and Moazenzadeh (2006) in a taxi driver study conducted in Iran agreed that hypertension and known coronary artery diseases risks were high amongst drivers who were employed for a period of more than 4 years. The study also found that obesity, gastrointestinal diseases, fatigue, musculoskeletal system complaints, sensory complaints including haemorrhoids were higher than in general population. Dahl *et al*. (2009) in a study conducted in Teamster in the United States found drivers working for Motor Freight Carriers were obese. The study found that half of the drivers had a BMI in the obese range which was double compared to the general population. Bigert *et al*. (2003) found that obesity was a concern amongst drivers who were frequently hospitalised with obstructive sleep apnoea and reported sleeping while driving. Another study conducted amongst taxi drivers in Taipei, Taiwan, confirmed that taxi drivers had suffered from systemic inflammatory diseases (Chen *et al*. 2005). In the current findings, obesity, elevated BP, fatigue and known diabetes mellitus were reported by participants. These are health risk factors associated with CV diseases.

Chen *et al*. (2005) further stated that high prevalence of lower back pain (LBP) could be associated with long driving time, physical and psychosocial factors, while similar results were reported by Alperovitch-Najenson *et al*. (2010) in professional bus drivers in Israel. Furthermore, AL-Dubai *et al*. (2012) in a study conducted in Malaysia showed that there were high levels of low-back pain and found that taxi drivers were reluctant to disclose LBP because of fear of loss of income and unemployment should they be unable to work. In the current study, the respondents also reported that they experience LBP.

A study amongst South Asian taxi drivers by Gany *et al*. (2014) indicates that despite available evidence of the effect of driving on health, no intervention is targeted to this population cohort. The current study indicated that all respondents were men. Peerson and Saunder (2009) lament that men’s health issues are not adequately addressed. The current study demonstrated that there are health risk issues amongst taxi drivers, and these are inadequately addressed. There is therefore a gap in the practice regarding adequate provision of health to taxi drivers. There is paucity of literature in the South African context regarding the health of drivers including taxi drivers. These could be associated with the society being multicultural and most cultures remaining patriarchal wherein provision of health services is lacking.

## Research design

A quantitative, descriptive and exploratory study was used. The population of the study was all taxi drivers in Tshwane who were willing to give a written consent, to have their BP, weight and height monitored and responded to a questionnaire. Convenient sampling was done at any taxi rank within Tshwane. Data were statistically analysed using STATA version 11, and a statistician was consulted to ensure statistical interpretation.

### The context of the study

The study emanated from evidence-based teaching and learning of Occupational Health Nursing in B.Tech Nursing at the Tshwane University of Technology. Taxis are the most accessible transport for majority of those who need to use public transport. Taxi drivers, who spend most of the time in their cars, were found to be working abnormal hours, which may result in fatigue and may impact their health status (Ramukumba, 2012).

### Population and sampling

The targeted population of the study was all taxi drivers who were found at taxi ranks in Tshwane. The accessible population was all taxi drivers whose taxis were awaiting their turn to load for different venues. Non-probability convenient sampling was done. The sample comprised 69 respondents who gave an informed consent and were willing to be assessed for health by students at various taxi ranks in Tshwane. Each student was assigned to approach at least four taxi drivers at various taxi ranks. Permission to collect data was given by the local taxi marshals stationed at various taxi ranks.

### Data collection

Data collection was done through self-report; the instrument used was a questionnaire. The questionnaire addressed record of physical measurements which included BP, weight, height and BMI that was calculated, and waist circumference measured at the taxi ranks. Data collection procedure was done as follows:

The students (B.Tech Community Nursing) were given four questionnaires, a bathroom scale which was calibrated and tape measure including a sphygmomanometer to use.

Data were collected at taxi ranks in Tshwane by students who were skilled in health assessment techniques. The time the students used to collect the data was credited to them as experiential learning, and they were only briefed to collect data within Tshwane borders and to go to taxi ranks in pairs.

### Data treatment

Data were processed and cleaned through Microsoft Excel. All incomplete questionnaires were discarded.

## Results of the study

After checking for completeness of the questionnaire, the realisation of data was that there were 69 (*n* = 69) respondents and all were males.

### Demographic results

Table 1 shows the age distribution of the respondents in the study.

**TABLE 1:** Age.

Variable (age)	*n*	%
21–30 years	9	13
31–40 years	33	48
41–50 years	17	25
51–60 years	5	7
61–70 years	5	7

**Total**	**69**	**100**

### Health status factors

**FIGURE 1:**
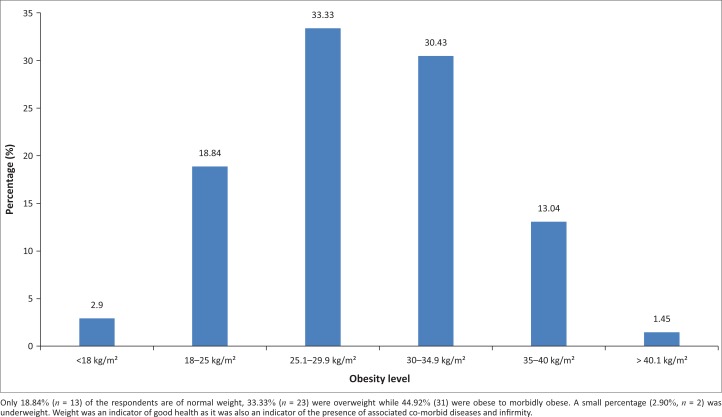
Obesity levels.

Figure 1 shows obesity levels amongst taxi drivers in the study.

### Respondents on use of medications

Only 15 of the 69 respondents indicated that they were on medication. These included 8.69% (*n* = 6) who were on hypertensive, 1.45% (*n* = 1) who was on diabetic and 7.24% (*n* = 5) who were on joint problem medications, while 79.71% (*n* = 55) were not on any medications.

### Musculoskeletal related problems

In this study, 33.33% (*n* = 23) respondents indicated that they have back problems. A further 18.85% (*n* = 13) indicated that they have discomforts which were not specified and the rest did not indicate anything related to the musculoskeletal problems.

### Lifestyle behaviours

#### Use of harmful substances

Of all the participants, 79.71% (*n* = 55) reported to have the harmful habits of cigarette smoking, dagga and alcohol intake; while 20.2% (*n* = 14) took only one, others used them all. Some participants also reported being on hypertensive medications and verbalised to be also drinking alcohol and driving, which was a concern. Meanwhile, some respondents were receiving treatment for diabetes mellitus and found to be drinking, smoking and driving. Lastly, a respondent expressed that he took alcohol daily while in transit. Furthermore, a respondent on antiretroviral therapy also smoked cigarette and dagga. All these conditions reportedly lowering the immune system.

#### Condom use

More than half of the respondents (53.62%, *n* = 37) indicated that they do not use condom protection during sexual intercourse, while the rest indicated they use condoms sometimes and not all the times which was a concern.

### Eating behaviours

Almost three quarters (81.16 %, *n* = 56) reported eating from taxi vendors or other fast food outlets.

#### Exercise

There was a general lack of exercise, with 77.79% (*n* = 53) participants reporting not doing exercise at all.

## Ethical consideration

Permission was sought from the taxi management, and data were collected in 2010.

### Informed consent

Written informed consents were signed after the students read the information leaflet for participants and explained them of the aim and objectives of data collection. The principle of privacy was met by ensuring that no passengers were in the vehicle during anthropometric measurements monitoring. Confidentiality was ensured by giving the participants immediate feedback, and those with suggestive deviations from normal values were referred to local clinics for further treatment and health information on lifestyle modification.

### Potential benefits and hazards

The potential benefits are that the results would be made available to the Department of Health to reach out to the health needs of this cohort group, and those who were found to have health problems were referred to their local clinics for early diagnosis and treatment. Results mitigate for further research in the taxi industry on health assessment, sleep deprivation and fatigue.

### Recruitment procedures

The taxi drivers were recruited through the taxi marshals, and none of them were coerced to be part of the study. They were informed that they could withdraw from the study at any time and no punitive measures will be taken against them.

### Data protection

Raw data will be kept at Adelaide Tambo School of Nursing Science in Tshwane University of Technology (TUT), Pretoria campus, for a period of 5 years.

### Validity

The instruments that were used by the students were pretested at a particular taxi rank to ensure that it measured was it was supposed to measure.

### Reliability

The anthropometric measurement amongst the taxi drivers will yield the same results if it can be repeated amongst taxi drivers in another province or setting, and therefore can be generalised amongst any group of taxi drivers.

## Discussion of results

### Outline of the results

Potgieter *et al*. (2012) in a study in Western Cape, South Africa, reported that out of 225 of their respondents the majority (223) were males (99%), while in the current study all the 69 participants (100%) were males aged between 30 and 49. Potgieter *et al*.’s (2012) study found that taxi drivers had knowledge and awareness of unsafe sex practices and the need to use condoms but did not actually use them. Respondents in the study expressed that it was only necessary to use condoms when with ‘taxi queens’ and not with ‘young girls’, raising the fear that this practice could spread HIV and AIDS. Also the study revealed that taxi drivers take alcohol or drugs which have far-reaching implications of leading to unsafe sex practices because of loss of judgement. Taravella (2005) further expressed that taxi drivers are at risk of HIV and AIDS because of lack of knowledge of condom use and risky sexual behaviours. Similarly, the current results demonstrated the use of alcohol and drugs and indiscriminate use of condoms. Moreover, these risky practices call for intensifying men’s health prevention and promotion strategies to address these vulnerable groups.

### Practical implications reaffirm the importance of the study

Most taxi drivers were of early adulthood to middle age. Taxi drivers have proven from other studies in various countries to be a vulnerable group, leading an unhealthy lifestyle in general with consequential health effects. In this study, evidence has shown that taxi drivers were obese to morbidly obese. They reported to be eating anything they could grab on the road. This behaviour entails unhealthy imbalance diet or poor eating behaviour. The consequences of obesity are that participants were exposed to conditions such as CV disease, hypertension, type 2 diabetes mellitus and other morbidity and co-morbidity (Bray 2009). Furthermore, drivers led a sedentary lifestyle evidenced by lack of exercise which posed to a health risk. Commercial truck drivers experience all four occupation related risk factors for obesity: sedentary lifestyle, poor opportunities for regular, healthy meals, irregular hours and sleep schedules (Beechy, Oglesby & Ahern 2014). This was confirmed in this study, where some taxi drivers indicated that they are on chronic medications for HBP, diabetes and joint pain. In a study conducted in Singapore by Lim and Chia (2015), similar results were found suggesting that a high proportion of the taxi drivers were obese and self-reported diagnosed with hypertension, diabetes mellitus. These two conditions could lead to fatigue, predisposing taxi drivers and their passengers to risk of accidents. The physiological assessment also found that drivers had elevated HBP and sugar level without their knowledge. Implication of this identification of health risks amongst taxi drivers is that interventions could be planned to improve the well-being of taxi drivers and avoid lifestyle illnesses that could be prevented and effectively managed which if unknown could be life-threatening for drivers, commuters and other road users.

There were reports of joint back problems and discomforts. Chan and Chen (2009) suggest that there is an increased association between obesity and musculoskeletal pain that makes vulnerability to fracture risks to escalate. Furthermore, BMI directly reflects in the drivers’ level of activity and overall functional capacity. These findings imply the need to motivate for the provision of occupational health medical surveillance screenings to assess wellness and opportune them to have early diagnosis and treatment of non-communicable diseases. This move could be beneficial to the community at large because of the fact that taxis are the highest mode of transport from local townships to the central business district area.

Lifestyle behaviours of use of harmful substance were reported in this study. Amongst other lifestyle behaviours reported were unsafe sexual practices, eating behaviours and the lack of exercise. The implications therefore should be campaigns to improve taxi drivers’ health awareness and the need for health promotion strategies that cannot be underestimated as part of the strategy to reduce road accidents injuries and their impact on the burden of disease in South Africa.

### Limitations of the study

The study was contextual and therefore could not be generalised to the entire population of the taxi industry.

### Recommendations emerging out of the current results

All taxi drivers should undergo pre-placement screening and periodic health assessment for possible fitness for task assessment. This could be enforced by law and regulation. Stress as a lifestyle challenge was not clearly defined. The need for accessible and available occupational health services is mandatory for this cohort group.

### Recommendations

Men’s health programmes should be intensified across the world as the part of men’s awareness of diseases seems to be lacking (White 2006).

There should be a mechanism of law enforcement ensuring that only healthy drivers who undergo periodic checks are allowed to drive taxis. Men’s health-friendly clinic needs to be advocated for in Tshwane.

## Conclusions

This section provides a brief conclusion that restates the objectives, the research design, the results and their meaning.

The objective of the study was to assess the health profile and the influence of driving on the taxi drivers in Tshwane. It is with no reservation to state that driving has consequential effects on the health of the driver, especially in the taxi industry, which results in prolonged driving as it was revealed in this study. Quantitative exploratory design was used where convenience sampling was withdrawn from varying taxi ranks in Tshwane wherein participation was voluntary. The study results revealed that taxi drivers are sick and have a gap of knowledge of what the meaning of health and wellness was. Furthermore, taxi drivers spent most of their time behind the wheel carrying passengers wherein they do not even have the skill of people management, which results in stress to carry their tasks. It was also revealed that taxi drivers did not have access to health screening facilities. The taxi industry is one of the vulnerable male-dominated groups. Especially, taxis form part of the largest public transport and therefore an urgent need of intersectoral collaboration is required, involving Department of Transport, Department of Health and Department of Labour for creating a work environment that can ensure promotion of health amongst taxi drivers. Also men’s health clinics need to be put in place; there should be mobile clinics on site and calendar dates for massive campaigns of health screening, and other health services at taxi ranks.
